# The effect of peer education compared to education provided by healthcare providers on premenstrual syndrome in high school students: A social network‐based quasi‐experimental controlled trial

**DOI:** 10.1002/npr2.12305

**Published:** 2022-11-21

**Authors:** Farzaneh Babapour, Forouzan Elyasi, Monirolsadate Hosseini‐Tabaghdehi, Jamshid Yazdani‐Charati, Zohreh Shahhosseini

**Affiliations:** ^1^ Student Research Committee Mazandaran University of Medical Sciences Sari Iran; ^2^ Sexual and Reproductive Health Research Center Mazandaran University of Medical Sciences Sari Iran; ^3^ Department of Midwifery, Health Reproductive Research Center, Sari Branch Islamic Azad University Sari Iran; ^4^ Health Sciences Research Center, Addiction Institue Mazandaran University of Medical Sciences Sari Iran

**Keywords:** adolescent, education, healthcare providers, Peer group, premenstrual syndrome

## Abstract

**Aim:**

This study aimed to compare the effect of peer education and education provided by healthcare providers on PMS in high school students.

**Materials and Methods:**

This quasi‐experimental non‐randomized controlled trial with a three‐armed parallel design was performed on 90 students allocated in three groups, namely, education by peer (intervention group 1 = 30), education by a healthcare provider (intervention group 2 = 30), and a control group (*n* = 30). The primary outcome was a change in the score of PMS, and the secondary outcomes were changes in the general health score and the frequency of premenstrual dysphoric disorder (PMDD). Education about PMS management was held in WhatsApp messenger in six sessions (two sessions per week) for both intervention groups. All three groups received routine school counseling. The researchers applied repeated‐measures ANCOVA, McNemar, and post‐hoc Bonferroni tests.

**Results:**

Education in intervention group 1 (Partial Eta Squared = 0.67, *p* < 0.0001) and intervention group 2 (Partial Eta Squared = 0.82, *p* < 0.0001) significantly reduced the PMS score compared to the control group. In addition, the change in general health score in the intervention groups compared to the control group showed the effectiveness of the intervention (*p* < 0.001). Education did not significantly reduce PMDD frequency in the intervention groups compared to the control group (*p* > 0.05).

**Conclusion:**

This study suggests education by peers and healthcare providers effects on PMS and general health in adolescents. It suggested that the effectiveness of these approaches be investigated in other adolescents' health conditions.

## Abstract 2

This study was conducted in two stages. The first step involved identifying students with moderate to severe PMS using Premenstrual Symptoms Screening Tool. Then, 96 eligible students, who were willing to participate in the study, entered the second stage of the study. Among them, six students from a school were selected as peer educators, and 30 students from the same school were included in the intervention groups. From the rest of the eligible students, 30 were randomly selected as the second intervention group and 30 students as the control group.

## Graphical abstract

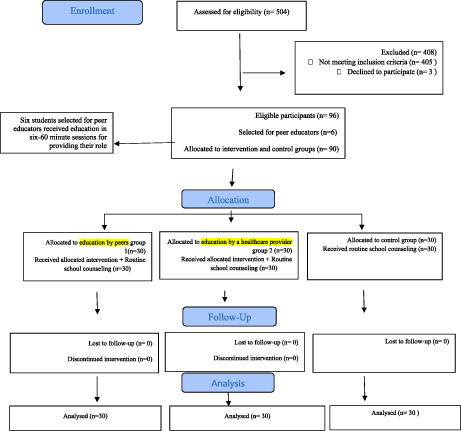

## 1 INTRODUCTION

Adolescence is one of the most valuable periods of one's life and the beginning of physical, psychological, and social changes that affect the performance of individuals in adulthood.^1^, ^2^ Menstruation is a critical phenomenon in adolescence. Although it is known as a physiological and natural phenomenon, premenstrual syndrome (PMS) is one of the common concerns.^2^ This syndrome is a neuropsychiatric disorder with biological, psychological, and social parameters.^3^

Symptoms of this disorder periodically appear as a combination of physical, psychological, and behavioral changes in the luteal phase of the menstrual cycle and can lead to disruption in a person's daily activities.^4^ American College of Obstetricians and Gynecologists has published some criteria for detecting PMS, including affective or somatic symptoms like anxiety, irritability, depression, mood swings, sleep disorders, fatigue, changes in sexual desire, breast tenderness, weight gain, headache, change in appetite, and aches and pains in the body.^5^ The symptoms are mild to moderate in most cases; however, about 5%–8% of women suffer from severe PMS.^6^ When the symptoms are severe and annoying and disturb one's life, it is called premenstrual dysphoric disorder (PMDD).^7^

Existing literature has shown that the prevalence of PMS is between 40% and 80% in the world.^8^, ^9^ Despite the high prevalence of PMS in adolescents, managing the symptoms of this syndrome still is challenging.^10^ There are various medical and non‐medical interventions for its management.^11^ They are including pharmaceutical treatments,^12^ complementary medicine,^13^ psychological counseling,^14^ diet changes,^15^ and surgical treatment.^16^ However, researchers emphasize choosing educational approaches as the first step in managing adolescent PMS.^17^, ^18^, ^19^ Evidence shows that peer group is one of the effective ways to educate adolescents to improve health‐related behaviors. The purpose of peer education is to develop knowledge, attitudes, and proper health behaviors by those who are not professionally trained but have common experiences. Peers can include family members, friends, and classmates.^20^ A trained peer can interact with others, effectively convey information to others, and communicate with healthcare professionals and other adolescents by promoting empathy and trust.^21^ Given the effect of PMS and its consequences on adolescents' health, this study aimed to compare the effect of peer education and the education by healthcare providers on PMS among high school students.

## 2 METHOD

### 2.1 Design and study setting

The trial protocol is reported elsewhere.^22^ The present study is a quasi‐experimental non‐randomized controlled trial with a three‐armed parallel design, including intervention group 1 (peer education), intervention group 2 (education by a healthcare provider), and a control group. We applied the Transparent Reporting of Evaluations with Nonrandomized Designs (TREND) Statement.^23^

### 2.2 Sample size

The pilot method was used to determine the volume of the study. Analysis of the information obtained from half of the participants, 45 students (15 students in each group) participating in the study. According to the Confidence Coefficient: 95%, Power: 80%, Effect Size (ES) = 0.8, and by considering an attrition rate of 25%, the sample size in each group was calculated, 30 students as follows:

$$n=\frac{2*{\left(\;z1-\frac{\alpha }{2}+z\;1-\beta \right)}^{2}}{E.S^{2}}=\frac{2*{\left(\;1.96+0.84\right)}^{2}}{{\left(0.8\right)}^{2}}=30$$

### 2.3 Inclusion and exclusion criteria

The 11th‐grade single students with regular menstruation (interval of 21–35 days) and moderate to severe PMS were enrolled. The exclusion criteria encompassed students with chronic illnesses (such as irritable bowel syndrome, coagulation disorders, and cancer), professional athletes, and students who used alternative therapies such as traditional medicine to control the PMS symptoms. Moreover, those who took hormonal treatments (e.g., estrogen, progesterone, their compounds, or antidepressants) and those who have experienced traumatic events, such as severe accidents, the death of family members (father, mother, siblings), and parental divorce over the past 6 months were excluded.

### 2.4 Outcomes

The primary outcome was a change in the PMS score in the intervention groups compared to the control group. Besides, changes in general health scores and the frequency of PMDD were considered secondary outcomes.

### 2.5 Recruitment

This study was conducted in two stages from December 2020 to September 2021 in Sari, northern Iran. The first step involved identifying students with moderate to severe PMS using Premenstrual Symptoms Screening Tool. By referring to six purposefully selected schools in Sari (among the 14 high schools), the census sampling method was employed to assess the PMS severity of 504 students. At this stage, 408 students were excluded from the study (405 students not meeting inclusion criteria and three students declined to participate). Then, 96 eligible students, who were willing to participate in the study, entered the second stage of the study. Among them, six students of a school with better power of speech and good communication skills (according to the school counselor and principal's viewpoint) were selected as peer educators, and 30 students from the same school were included in the first intervention group (education by peers). From the rest of the eligible students, with the assistance of a random number table, 30 students were selected as the second intervention group (education by a healthcare provider) and 30 students as the control group. In the follow‐up phase, no samples were lost in the groups.

### 2.6 Measurements

#### 2.6.1 Daily record of severity of problems (DRSP)

This questionnaire prospectively evaluates 30 PMS symptoms according to DSM‐V criteria in the form of physical, psychological, and social symptoms on a 4‐point Likert scale from 0 to 3. The scores range from 0 to 90, and a higher score indicates greater severity of PMS.^24^ The reliability and validity of the instrument were confirmed by Cronbach's alpha of 0.75 and Pearson correlation coefficient of 0.80.^25^ The intervention and control groups (*n* = 90) completed the questionnaire before and after the intervention (in two consecutive cycles), and the mean score was recorded as the PMS score.

#### 2.6.2 Premenstrual symptoms screening tool (PSST)

This 19‐question tool with two domains retrospectively examines the severity of PMS symptoms. The first domain includes 14 items related to psychological, physical, and behavioral symptoms, and the second domain (five items) measures the effect of these symptoms on the individual's life. Each question is rated on a 4‐point scale, i.e., not at all, mild, moderate, and severe, ranging from 0 to 3. To determine the mild to severe PMS, the researchers must simultaneously meet the following three conditions: there should be at least one item with moderate to severe from items 1 to 4, at least four items with moderate to severe from items 1 to 14, and one item in the second domain must be mild or severe. In addition, this instrument is used to screen the PMDD. It requires the following three conditions to be simultaneously detected: at least one severe item from items 1 to 4, at least four moderate or severe items from items 1 to 14, and at least one severe item in the last five items.^26^ This instrument's first and second domains have Cronbach's alpha of 0.96 and 0.91, respectively.^27^ Participants completed this instrument before and after the intervention.

#### 2.6.3 Goldberg general health questionnaire (GHQ)

This well‐known valid 28‐question instrument with four subscales is scored from 0 to 3 on the Likert scale. Scores range from zero to 84, and lower scores indicate better mental health.^28^ The reliability of the questionnaire was confirmed using three methods of test–retest (0.70), split‐half (0.93), and Cronbach's alpha (0.90). The concurrent validity of the instrument, as measured by Middlesex Hospital Questionnaire, was 0.55 (*p* < 0001). Subscale–total correlations, as another index of validity, were between 0.72 and 0.87.^29^ All the participants completed this questionnaire two times before and after the intervention.

### 2.7 Intervention

At first, the first researcher (a Master of Science midwifery student who worked under the supervision of the research team, i.e., a reproductive health professor and a psychiatrist) trained the six peer educators in person for six 60‐minute sessions (two sessions per week). The educational content included the anatomy and physiology of the female reproductive system, normal menstruation, related factors to PMS, its complications, and ways to manage it. Also, education about issues such as life skills, problem‐solving, critical thinking, decision‐making skills, creative thinking, and communication was provided. Then, the peer educators and a healthcare provider simultaneously provided their education for the intervention group 1 (30 students in three groups of 10) and intervention group 2 (a group of 30), respectively. In each group of 10, two trained students as peer educators led the sessions. The intervention was held in six 60‐minute sessions twice a week in WhatsApp messenger. The research team developed the content of the education sessions using the available resources. The protocol content as presented in Table 1 was approved by two professors of psychiatry and one professor of reproductive health at the university. In both intervention groups, the peer and the healthcare provider individually uploaded the pre‐prepared audio files on the education topics with the related PowerPoint file in each session and then allowed participants to ask possible questions. At the end of each session, the healthcare provider/peer asked questions about the topics and motivated the students to participate in the discussion. They were asked to send the answers to these questions to the interveners in the private conversation space. Finally, they ended the class by uploading the video file and the educational pamphlet of that session. The content of each training session was similar in both intervention groups. The control group did not receive any training, but similar to the two intervention groups, they had access to the school counselor and could refer to them if they had any questions.

**TABLE 1:** The content of training sessions in the intervention groups

Sessions	Content of the sessions
1	Introduction, statement of goals and rules of the sessions, explaining the menstrual disorders, talking to group members about menstrual disorders and initial measures to alleviate the symptoms from the participants' point of view, an overview of life skills including problem‐solving, critical thinking, decision‐making skills, creative thinking, the ability to be self‐aware
2	Introducing the female reproductive system and its different parts, defining puberty and its types, explaining hormonal changes and puberty‐initiating factors, describing physiological changes related to girls' puberty
3	A brief definition of menstruation, a complete description of menstruation and the physical condition due to it, defining the menstrual cycle and explaining different stages of the cycle, providing health advice about menstruation
4	Providing a summary of menstrual disorders, defining PMS, naming risk factors for this syndrome, providing a list of its different symptoms
5	Explaining the role of proper diet, exercise, and the factors influential in reducing the complications of PMS
6	Reviewing the fifth session by the peer educator and the participation of the participants, explaining the available pharmaceutical methods to eliminate and reduce the PMS symptoms, expressing the first step to control and manage PMS, providing simple solutions to reduce stress, providing a summary of the held sessions

### 2.8 Data analysis

The collected data were fed into SPSS version 18. Numerical variables were reported as mean and standard deviation, and categorical variables were presented as frequency and percentage. ANCOVA assessed the mean difference between the intervention and control groups in the primary outcome with repeated measures; the post‐hoc Bonferroni test controlled the increased risk of type I error due to multiple comparisons and compared the pairs with repeated‐measures tests with significant results. An independent *t*‐test was also used for secondary outcomes, and relative Eta Squared and Cohen's d were reported in the primary and secondary outcomes. McNemar's test was employed to describe the frequency of PMDD.

## 3 RESULTS

**FIGURE 1:**
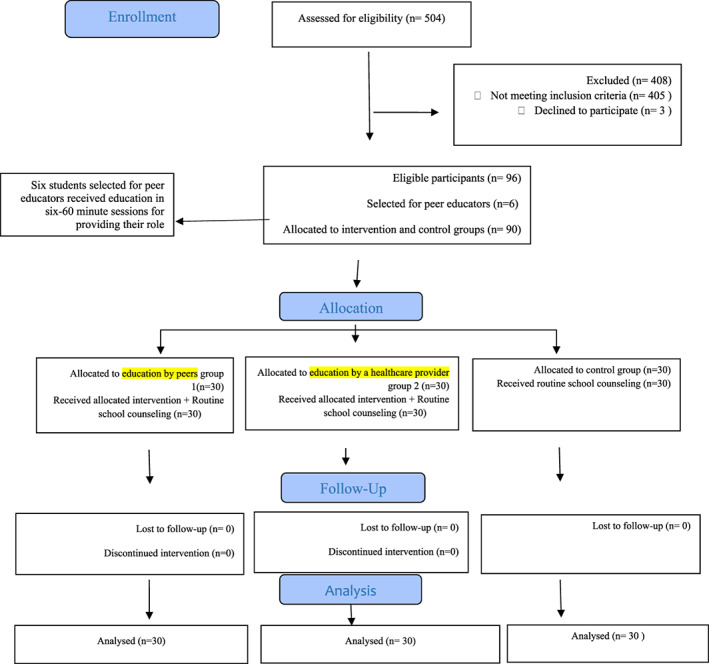
Flow diagram of the study

All 30 participants in each group completed the study and entered the analysis stage. The flow diagram of the study is presented in Figure 1. The mean age of participants in intervention groups 1, 2, and control groups were 16.5 ± 0.50, 16.47 ± 0.50, and 16.67 ± 0.47, respectively (*p* > 0.05). Table 2 illustrates other demographic characteristics.

**TABLE 2:** Participants' socio‐demographic characteristics

Variable	Intervention group 1	Intervention group 2	Control group	*p*‐Value
Age (years)^a^	16.50 ± 0.50	16.47 ± 0.50	16.67 ± 0.47	0.253
Menarche age (years)^a^	12.37 ± 1.35	12.93 ± 1.04	12.62 ± 0.99	0.174
Menstruation duration (day)^a^	5.73 ± 1.72	5.73 ± 1.53	6.27 ± 1.04	0.474
Residential area^b^				
City	26 (86.7)	23 (76.7)	21 (70)	0.336
Village	4 (13.3)	7 (23.3)	9 (30)	
Physical activity^b^				
No	6 (20)	10 (33.3)	10 (33.3)	0.769
Yes (irregular)	17 (56.7)	14 (46.7)	15 (50)	
Yes (regular)	7 (23.3)	6 (20)	5 (16.7)	
Dysmenorrhea^b^				
Yes	22 (73.3)	22 (73.3)	23 (76.7)	0.943
No	8 (26.7)	8 (26.7)	7 (23.3)	
Familial history of PMS^b^				
Yes	8 (26.7)	14 (46.7)	6 (20)	0.067
No	22 (73.3)	16 (53.3)	24 (80)	
Caffeine intake^b^				
Utmost one cup a day	24 (80)	24 (80)	24 (80)	1.000
At least two cups a day	6 (20)	6 (20)	6 (20)	

^a^ Presented as mean ± SD.

^b^ Presented as number (frequency).

### 3.1 Primary outcome: Premenstrual syndrome

As shown in Table 3, although there were similarities in the mean and standard deviation of the total score of PMS in the three groups before the intervention (*p* = 0.262), after interventions, significant differences were found among the three groups (*p* < 0.0001). The results of the ANCOVA repeated‐measure analysis and Bonferroni post‐hoc test showed after the intervention, the PMS score decreased in the intervention groups compared to the control group. Table 3 demonstrates that the effect size in the education by a healthcare provider group (Partial Eta Squared = 0.82, *p* < 0.0001) was more than the education by peers group (Partial Eta Squared = 0.67, *p* < 0.0001).

**TABLE 3:** Means and standard deviations of PMS score at the baseline and after the intervention

PMS score	Intervention group 1	Intervention group 2	Control group	*p*‐Value
Baseline	37.40 ± 7.13	37.94 ± 6.33	36.75 ± 6.44	0.262
Post intervention	29.28 ± 3.95	27.29 ± 2.68	34.88 ± 3.62	<0.0001
Effect size confidence interval	0.67 (1.0–27.11)	0.82 (1.0–37.23)	‐	‐
*p*‐Value	<0.0001	<0.0001	0.30	

### 3.2 Secondary outcomes: General health and premenstrual dysphoric disorder

Results (Table 4) illustrate that there were similarities in the mean and standard deviation of the total score of general health in the three groups before the intervention (*p* = 0.771), but after interventions, differences among the three groups were significant (*p* < 0.0001). It showed after the interventions, the mean score of general health significantly decreased in the education by peers group (Cohen's *d* = 0.25, *p* < 0.0001) and education by a healthcare provider group (Cohen's *d* = 0.37, *p* < 0.0001) compared to control group (Table 4). However, McNemar's test showed that intervention did not significantly reduce the frequency of PMDD in the two intervention groups compared to the control group (*p* > 0.05).

**TABLE 4:** Mean score and standard deviation of general health score, at the baseline, and after the intervention

General health score	Intervention group 1	Intervention group 2	Control group	*p*‐Value
Baseline	35.17 + 12.57	39.27 + 13.18	36.77 + 11.58	0.771
Post intervention	26.47 + 10.31	27.33 + 10.30	35.07 + 11.40	<0.0001
Effect size confidence interval	0.25 (0.81–0.31)	0.37 (0.93–0.19)	‐	‐
*p*‐Value	<0.0001	<0.0001	0.292	

## 4 DISCUSSION

The present study compared peer education and healthcare provider education regarding PMS in adolescents. The study illustrates that education in both intervention groups significantly reduced the PMS score compared to the control group. Similarly, several studies have shown that educational approaches can manage PMS symptoms.^17^, ^18^, ^19^ In this regard, Taylor et al.^30^ conducted a study to determine the effectiveness of peer group education on PMS in women. Consistent with this study, they showed that education by peers significantly reduces the PMS score. According to social identity theory, adolescents tend to be influenced by friends and classmates who have similar and common characteristics to others.^31^ Therefore, the intervention used in this study provided a suitable environment for using the friends' network among students with similar and common characteristics and could affect the PMS severity. This study reveals that if intervention programs to manage PMS focus on educating and empowering adolescents' favorite peers, they can influence their other peers.

In contrast to the present study, Sehati et al.^32^ investigated the effect of peer education on students' performance in iron supplementation. Their study showed that peer education was not effective in improving the weekly consumption of iron‐containing foods. Furthermore, Dabiri et al.^33^ compared the two health education methods (i.e., pamphlets and peers) on menstrual health performance; none affected the performance. The above findings may be because behavior requires more time to change.

The results of this study illustrate that the participants in the second intervention group which education provided by healthcare provider had lower mean PMS scores than peer education. Accordingly, Azizi et al.^34^ compared the effect of three education methods (i.e., peer, physician, and pamphlet) on students' knowledge about HIV infection prevention. The results revealed that the mean change in scores in the group trained by the physician was higher than those of the peer and pamphlet groups. The probable reasons can be the students' greater trust in the healthcare provider as a reliable source of information, the health practitioner's responsiveness and emphasis on the points students asked, and eliminating the ambiguities due to the comprehensive knowledge concerning the issue.

The results of the present study demonstrate that education significantly improved the general health of both intervention groups compared to the control group. In line of our study, some studies showed that health education by peers influences psychological, physical, and overall quality of life dimensions of adolescents.^35^, ^36^, ^37^ Correspondingly, peer cooperation imposes a less financial burden on the government and patients.^38^

## 5 STRENGTHS AND LIMITATIONS

One of the strengths of this study is that standard and valid questionnaires were used in this study, and the psychometric properties of these questionnaires have been assessed in Iran already.

Nonetheless, these results must be interpreted with caution, and a number of limitations should be borne in mind. Despite the non‐randomized sampling and potential selection bias, the study variables were not statistically different in the three groups at the beginning of the study. Randomized design with more samples in the future may provide more consistent evidence in this issue. Another limitation was the lack of blindness of the intervention; there is a possibility of performance bias. The other limitation of this project was that the questionnaires were self‐reports, so individual differences, tiredness, and boredom were the factors that could affect their responses. Therefore, the researcher emphasized the confidentiality of the information and explained the importance of their responses to decrease the error resulting from this limitation. Finally, that the participants may have had online searches and used other resources is another limitation of this study.

## 6 CONCLUSION

The study reveals that peer education can positively affect the PMS and general health in adolescent girls. It seems that applying this cost‐effective educational approach in schools and health centers can be an efficient step in promoting the health of adolescent girls and improving the quality of schools' health education programs. Finally, the results recommend that health policymakers with the participation of the education organization can act by using midwifery consultants as peer trainers. Although this study focused on PMS relief, this strategy is generally health promoting and can be applied to other adolescents' health conditions.

## AUTHOR CONTRIBUTIONS

All authors conceptualized and designed the study. Data were collected by FB and ZS. FB, ZS, and JYC performed the statistical analyses. ZS, FB, FE, MHT, and JYC collaborated in the interpretation of the results. ZS drafted the manuscript. ZS, FB, and MHT wrote the final version of the manuscript and revised it critically for important intellectual content. All authors read and approved the final manuscript.

## FUNDING INFORMATION

This project was fully supported and funded by Mazandaran University of Medical Sciences and the Student Research Committee of Mazandaran University of Medical Sciences, Grant number: 9188.

## CONFLICT OF INTEREST

The authors declare that they have no competing interests.

### ETHICAL APPROVAL

**Approval of the research protocol by an institutional review board:** This study was approved by the Medical Ethics Committee of the Mazandaran University of Medical Sciences (Ethical Code: IR.MAZUMS.REC.1396.9188). All methods were conducted in accordance with the ethical standards of the declaration of Helsinki. All participants (students and their parents) provided signed informed consent to participate in the study. Researchers obtained written informed consent from the students and their parents. To observe research ethics, the control group received all the educational files in WhatsApp messenger after collecting all the questionnaires.

**Informed consent**: NA.

**Registry and the registration no. of the study/trial:** Iranian Registry of Clinical Trials (IRCT), IRCT20150608022609N5. Registered 04 /08/ 2019, http://irct.ir/user/trial/20288/view.

**Animal studies**: N/A.

## Data Availability

Since our data contain sensitive personal information, it is forbidden to share these data with a third party without obtaining an additional written form of informed consent for information sharing. We did not obtain additional written consent for information sharing.

## References

[1] Shahhosseini Z , Simbar M , Ramezankhani A , Majd HA . Supportive family relationships and adolescent health in the socio‐cultural context of Iran: a qualitative study. Ment Health Fam Med. 2012;9(4):251–6. 24294300 PMC3721919

[2] Tadakawa M , Takeda T , Monma Y , Koga S , Yaegashi N . The prevalence and risk factors of school absenteeism due to premenstrual disorders in Japanese high school students—a school‐based cross‐sectional study. BioPsychoSocial Med. 2016;10(1):1–7. 10.1186/s13030-016-0067-3 PMC4845482 27118993

[3] Burkman RT . Berek & Novak's gynecology. JAMA. 2012;308(5):516–7.

[4] Moghadasi A , Abbasi M , Yousefi M , Kargarfard M . A comparison of prevalence of premenstrual syndrome symptoms between athlete and non‐athlete female students. Physiol Sport Phys Act. 2009;3:199–208.

[5] Obstetricians ACo, Gynecologists . ACOG practice bulletin: premenstrual syndrome. Int J Gynaecol Obstet. 2001;73:183–91.

[6] Yonkers KA , O'Brien PS , Eriksson E . Premenstrual syndrome. The Lancet. 2008;371(9619):1200–10. 10.1016/S0140-6736(08)60527-9 PMC3118460 18395582

[7] Di Scalea TL , Pearlstein T . Premenstrual dysphoric disorder. Psychiatr Clin. 2017;40(2):201–16. 10.1016/j.psc.2017.01.002 28477648

[8] Direkvand Moghadam A , Kaikhavani S , Sayehmiri K . The worldwide prevalence of premenstrual syndrome: a systematic review and meta‐analysis study. Iran J Obstet Gynecol Infertil. 2013;16(65):8–17.

[9] Ranjbaran M , Samani RO , Almasi‐Hashiani A , Matourypour P , Moini A . Prevalence of premenstrual syndrome in Iran: a systematic review and meta‐analysis. Int J Reprod Biomed. 2017;15(11):679–86. 29404529 PMC5780553

[10] Seedhom AE , Mohammed ES , Mahfouz EM . Life style factors associated with premenstrual syndrome among El‐Minia university students, Egypt. Int Sch Res Notices. 2013;617123:1–6.

[11] Yilmaz‐Akyuz E , Aydin‐Kartal Y . The effect of diet and aerobic exercise on Premenstrual Syndrome: randomized controlled trial. Nutr J. 2019;32:e18024.31242913

[12] Hantsoo L , Epperson CN . Premenstrual dysphoric disorder: epidemiology and treatment. Curr Psychiatry Rep. 2015;17(11):1–9. 26377947 10.1007/s11920-015-0628-3 PMC4890701

[13] Douglas S . Premenstrual syndrome. Evidence‐based treatment in family practice. Can Fam Physician. 2002;48(11):1789–97. 12489244 PMC2213956

[14] Kues JN , Janda C , Kleinstäuber M , Weise C . Internet‐based cognitive behavioural self‐help for premenstrual syndrome: study protocol for a randomised controlled trial. Trials. 2014;15(1):1–9. 25467540 10.1186/1745-6215-15-472 PMC4265499

[15] Abedian Kasgari K , Shahhosseini Z , Danesh M . Assessment of starch dietary regimen regarding pre‐menstrual syndrome among high school students in sari during 2007. J Maz Univ Med. 2008;18(65):19–27.

[16] Heliövaara‐Peippo S , Halmesmäki K , Hurskainen R , Teperi J , Grenman S , Kivelä A , et al. The effect of hysterectomy or levonorgestrel‐releasing intrauterine system on lower urinary tract symptoms: a 10‐year follow‐up study of a randomised trial. BJOG: Int J Obstet. 2010;117(5):602–9. 10.1111/j.1471-0528.2010.02505.x 20156209

[17] Ayaz‐Alkaya S , Yaman‐Sözbir Ş , Terzi H . The effect of health belief model‐based health education programme on coping with premenstrual syndrome: a randomised controlled trial. Int J Nurs Pract. 2020;26(2):e12816. 31985138 10.1111/ijn.12816

[18] Başoğul C , Aydın Özkan S , Karaca T . The effects of psychoeducation based on the cognitive‐behavioral approach on premenstrual syndrome symptoms: a randomized controlled trial. Perspect Psychiatr Care. 2020;56(3):515–22. 31788825 10.1111/ppc.12460

[19] Simsek Kücükkelepce D , Timur TS . The effects of health belief model‐based education and acupressure for coping with premenstrual syndrome on premenstrual symptoms and quality of life: a randomized‐controlled trial. Perspect Psychiatr Care. 2021;57(1):189–97. 32468669 10.1111/ppc.12546

[20] Ghasemi V , Simbar M , Rashidi Fakari F , Kiani Z . The effect of peer education on health promotion of Iranian adolescents: a systematic review. Int J Pediatr. 2019;7(3):9139–57.

[21] Lotfi Mainbolagh B , Rakhshani F , Zareban I , Alizadeh Sivaki H , Parvizi Z . The effect of peer education based on health belief model on nutrition behaviors in primary school boys. J Res Health. 2012;2(2):214–26.

[22] Babapour F , Elyasi F , Yazdani‐charati J , Shahhosseini Z . A comparison between the effects of school‐based education programs provided by peer group versus health practitioners on premenstrual syndrome in adolescents: a protocol for a non‐masked clinical trial. Nursing Open. 2021;8(5):2901–8. 33715300 10.1002/nop2.858 PMC8363407

[23] Haynes AB , Haukoos JS , Dimick JB . TREND reporting guidelines for nonrandomized/quasi‐experimental study designs. JAMA Surg. 2021;156(9):879–80. 33825826 10.1001/jamasurg.2021.0552

[24] Henz A , Ferreira CF , Oderich CL , Gallon CW , Castro JRS , Conzatti M , et al. Premenstrual syndrome diagnosis: a comparative study between the daily record of severity of problems (DRSP) and the premenstrual symptoms screening tool (PSST). Rev Bras Ginecol Obstet. 2018;40:20–5. 29132173 10.1055/s-0037-1608672 PMC10467366

[25] Sharifi F , Simbar M , Mojab F , Majd HA . Comparison of the effects of Matricaria chamomila (chamomile) extract and mefenamic acid on the intensity of premenstrual syndrome. Complementary therapies in clinical practice. 2014;20(1):81–8. 24439651 10.1016/j.ctcp.2013.09.002

[26] Steiner M , Peer M , Palova E , Freeman EW , Macdougall M , Soares CN . The premenstrual symptoms screening tool revised for adolescents (PSST‐A): prevalence of severe PMS and premenstrual dysphoric disorder in adolescents. Arch Womens Ment Health. 2011;14(1):77–81. 21271266 10.1007/s00737-010-0202-2

[27] Yen J‐Y , Chang S‐J , Long C‐Y , Tang T‐C , Chen C‐C , Yen C‐F . Working memory deficit in premenstrual dysphoric disorder and its associations with difficulty in concentrating and irritability. Compr Psychiatry. 2012;53(5):540–5. 21821238 10.1016/j.comppsych.2011.05.016

[28] Vergara‐Moragues E , González‐Saiz F . Predictive outcome validity of general health questionnaire (GHQ‐28) in substance abuse patients treated in therapeutic communities. J Dual Diagn. 2020;16(2):218–27. 31608803 10.1080/15504263.2019.1674465

[29] Taghavi S . Validity and reliability of the general health questionnaire (ghq‐28) in college students of shiraz university. J Psychol. 2002;5(4):381–98.

[30] Taylor D . Effectiveness of professional–peer group treatment: symptom management for women with PMS. Res Nurs Health. 1999;22(6):496–511. 10625865 10.1002/(sici)1098-240x(199912)22:6<496::aid-nur7>3.0.co;2-2

[31] Turner G , Shepherd J . A method in search of a theory: peer education and health promotion. Health Educ Res. 1999;14(2):235–47. 10387503 10.1093/her/14.2.235

[32] Sehhati Shafai F , Mohammad Alizadeh CS , Ebrahimi M , Salmani R . The effects of peer education on girl students' knowledge and performance about iron deficiency and iron supplementation. J Maz Univ Med. 2013;22(1):223–33.

[33] Dabiri F , Abedini S , Shahi A , Kamjoo A . The effect of different methods of health education on knowledge, attitudes and practice of female students regarding menstrual hygiene in Bandar Abbas (2006). HMJ. 2009;12(4):271–9.

[34] Azizi A , Amirian F , Amirian M . The comparison effect of three educational methods about HIV prevention by peer physician and pamphlet diffusion on knowledge of girl's students in high schools. Hayat. 2008;14(1):5–12.

[35] Diao H , Pu Y , Yang L , Li T , Jin F , Wang H . The impacts of peer education based on adolescent health education on the quality of life in adolescents: a randomized controlled trial. Qual Life Res. 2020;29(1):153–61. 31562569 10.1007/s11136-019-02309-3

[36] Madani A , Alizade A , Ghanbarnejad A , Aghamolaei T . Effect of peer education on health promoting behaviors of junior high school students. Iran J Health Educ Health Promo. 2015;3(2):105–15.

[37] Sistani N , Khoi M , Taghdisi MH . Promoting knowledge, attitude and practices (KAP) of the mothers in their Girls' pubertal health based on peer education approach. J Babol Univ Med Sci. 2010;11(6):33–9.

[38] Khalajabadi Farahani F , Ebadifar AF . Comparing the effect of peer‐led versus adult‐led AIDS education on knowledge, attitude and self‐efficacy of female students in high schools in 4th region of education ministry in Tehran, using socio‐cognitive theory, 2002–2003. 2004.

